# Isolevuglandins Promote Mitochondrial Dysfunction and Electrophysiologic Abnormalities in Atrial Cardiomyocytes

**DOI:** 10.3390/cells13060483

**Published:** 2024-03-09

**Authors:** Tuerdi Subati, Zhenjiang Yang, Matthew B. Murphy, Joshua M. Stark, David Z. Trykall, Sean S. Davies, Joey V. Barnett, Katherine T. Murray

**Affiliations:** 1 Departments of Medicine and Pharmacology, Vanderbilt University School of Medicine, Nashville, TN 37232, USA; tuerdi.subati@vumc.org (T.S.); zhenjiang.yang@vanderbilt.edu (Z.Y.); matthew.b.murphy@vanderbilt.edu (M.B.M.); josmstar@uthsc.edu (J.M.S.); dtrykall@gmail.com (D.Z.T.); sean.davies@vanderbilt.edu (S.S.D.); joey.barnett@vanderbilt.edu (J.V.B.); 2 Division of Clinical Pharmacology Room 559, Preston Research Building, Vanderbilt University School of Medicine, 2220 Pierce Avenue, Nashville, TN 37232, USA

**Keywords:** Isolevuglandins, oxidative stress, lipid dicarbonyl, atrial HL-1 cells, mitochondria, electrophysiology, atrial fibrillation

## Abstract

Atrial fibrillation (AF) is the most common sustained cardiac arrhythmia, yet the cellular and molecular mechanisms underlying the AF substrate remain unclear. Isolevuglandins (IsoLGs) are highly reactive lipid dicarbonyl products that mediate oxidative stress-related injury. In murine hypertension, the lipid dicarbonyl scavenger 2-hydroxybenzylamine (2-HOBA) reduced IsoLGs and AF susceptibility. We hypothesized that IsoLGs mediate detrimental pathophysiologic effects in atrial cardiomyocytes that promote the AF substrate. Using Seahorse XFp extracellular flux analysis and a luminescence assay, IsoLG exposure suppressed intracellular ATP production in atrial HL-1 cardiomyocytes. IsoLGs caused mitochondrial dysfunction, with reduced mitochondrial membrane potential, increased mitochondrial reactive oxygen species (ROS) with protein carbonylation, and mitochondrial DNA damage. Moreover, they generated cytosolic preamyloid oligomers previously shown to cause similar detrimental effects in atrial cells. In mouse atrial and HL-1 cells, patch clamp experiments demonstrated that IsoLGs rapidly altered action potentials (AP), implying a direct effect independent of oligomer formation by reducing the maximum Phase 0 upstroke slope and shortening AP duration due to ionic current modifications. IsoLG-mediated mitochondrial and electrophysiologic abnormalities were blunted or totally prevented by 2-HOBA. These findings identify IsoLGs as novel mediators of oxidative stress-dependent atrial pathophysiology and support the investigation of dicarbonyl scavengers as a novel therapeutic approach to prevent AF.

## 1. Introduction

Atrial fibrillation (AF) constitutes a major public health problem, causing substantial morbidity including stroke, heart failure, and dementia, as well as death [1]. Currently, available pharmacologic therapies are often ineffective in preventing AF, indicating that key molecular pathways in AF pathogenesis remain elusive. Inflammation is implicated in the genesis of AF, and oxidative injury plays a key role in the detrimental effects of inflammation-related diseases. Elevated oxidative stress due to the excessive generation of reactive oxygen species (ROS) is a key pathophysiological feature of multiple AF risk factors such as hypertension [2]. Mitochondria are instrumental in maintaining normal mechanical and electrical activity of cardiomyocytes, as they constitute over 30% of the cardiomyocyte volume and are responsible for generating over 90% of cellular ATP through oxidative phosphorylation [3]. Mitochondria are also a major source of ROS in cardiomyocytes, and increasing evidence indicates a correlation between ROS damage, mitochondrial dysfunction, and cardiomyocyte remodeling in AF [4,5,6,7,8,9].

One of the most vulnerable sites to injury by ROS are polyunsaturated fatty acids in the plasma membrane and circulation. Lipid peroxidation generates multiple byproducts that contain reactive aldehyde and/or ketone groups, including malondialdehyde and 4-hydroxy-nonenol. These products can adduct proteins, DNA, and lipids to modify molecular function, and they are potentially more injurious than ROS, given their longer half-life and uncharged nature that enables membrane penetration. The most reactive lipid oxidation products identified to date are dicarbonyl compounds known as isolevuglandins (IsoLGs), and they are increased in the early stages of numerous diseases linked to oxidative stress [10]. The development of small molecule lipid dicarbonyl scavengers such as 2-hydroxybenzylamine (2-HOBA) has enabled preclinical studies demonstrating that IsoLGs are responsible for a major component of oxidative stress-related injury [10,11,12]. In addition, mitochondrial IsoLGs have been shown to promote vascular oxidative stress, while mitochondria-targeted scavenging of IsoLGs reduces mitochondrial dysfunction and hypertension [13].

In a recent study, AF susceptibility in hypertensive mice was associated with the appearance of both IsoLG adducts and natriuretic peptide-derived preamyloid oligomers in the atrial myocardium, all of which were prevented by 2-HOBA treatment [11]. It is recognized that IsoLGs accelerate the process of oligomer formation for amyloid-forming proteins, and oligomers have been shown to cause pro-arrhythmic metabolic and electrophysiologic effects in atrial myocytes [14]. Whether IsoLGs are independent pathologic mediators in the absence of oligomer generation is not known.

Here, we hypothesized that IsoLGs are direct mediators of oxidative stress-induced atrial cardiomyocyte dysfunction. Employing a multifaceted approach, we provide evidence for a mechanistic link for IsoLGs to cause both mitochondrial and electrophysiological remodeling in atrial cells. These results support the therapeutic potential of targeting reactive lipid dicarbonyl mediators such as IsoLGs for the prevention of AF. 

## 2. Materials and Methods

An expanded version of the Methods is provided in the Supplementary Materials.

### 2.1. Mouse Atrial Cardiomyocyte Isolation

Male C57BL/6J mice were obtained from Jackson Laboratory and studied at 3 to 4 months of age. Mice were maintained under 12:12 h light-dark cycles with standard chow and water ad libitum. All animal procedures were approved by the Vanderbilt Institutional Animal Care and Use Committee with care in accordance with the Guide for the Care and Use of Laboratory Animals, US Department of Health and Human Services. Mouse atrial cardiomyocytes were isolated using collagenase digestion [14].

### 2.2. Cell Culture

Atrial HL-1 cells [15] were cultured on fibronectin/gelatin-coated dishes in supplemented Claycomb Medium as described previously [16]. Mouse fibroblast (*Ltk^−^*) cells that stably express *KCNA5*, representing the rapidly activating sustained outward K^+^ current I_sus_, were also cultured for electrophysiologic experiments [14].

### 2.3. Cytotoxicity and Metabolic Flux Assays

Atrial HL-1 cells were treated with different concentrations of IsoLGs for 2 h. Cytotoxicity of IsoLGs was determined by measuring cellular ATP levels with an ATPlite assay (Perkin Elmer, Waltham, MA, USA) according to the manufacturer’s instructions [11].

The Seahorse XFp Cell Mito Stress Test (Agilent, Santa Clara, CA, USA) was used to analyze oxygen consumption rates (OCRs) and various bioenergetic parameters in atrial HL-1 cells in response to different concentrations of IsoLG for 2 h [14]. OCR was measured with a Seahorse XFp Analyzer following sequential injections of oligomycin, carbonyl cyanide p-trifluoromethoxyphenylhydrazone (FCCP), and antimycin A/rotenone. Cells were stained with Hoechst 33342 and imaged, with cell numbers in each well used to normalize all OCR parameters.

### 2.4. Mitochondrial Measurements

To assay mitochondrial membrane potential (Δψm), atrial HL-1 cells were treated with IsoLG for 2 h using a range of concentrations, followed by incubation with tetramethylrhodamine methyl ester (TMRM; to identify functional mitochondria in a membrane potential manner), Mitotracker Green (to label functional mitochondria irrespective of membrane potential), and Hoechst 33342 (to visualize nuclei), with live cell imaging using either the ImageXpress Micro XLS System (Molecular Devices, San Jose, CA, USA) for high content analysis or a Zeiss 880 confocal microscope (Carl Zeiss Microimaging, Inc., Oberkochen, Germany). As a positive control, a cohort of cells was treated with FCCP.

For mitochondrial DNA (mtDNA) content, total DNA was isolated from atrial HL-1 cells as described previously [17]. Mitochondrial DNA content was estimated from the ratio of mitochondrial DNA to nuclear DNA by quantitative polymerase chain reaction (qPCR) [18]. Data analysis was performed using the 2^−ΔΔCT^ method [19]. Primers are available in the Supplementary Materials (Table S7).

### 2.5. Detection of Cytosolic and Mitochondrial Superoxide Production

The fluorescent dye dihydroethidium (DHE) was employed to assess intracellular superoxide (O_2_^•−^) production in atrial HL-1 cells in response to IsoLG treatment, with live cell images acquired using confocal microscopy. To assess mitochondrial O_2_^•−^ production during treatment with IsoLGs, cells were incubated with MitoSOX Red, a mitochondrial-targeted fluorescent indicator of O_2_^•−^, and live cell images were captured by confocal microscopy.

### 2.6. Protein Carbonylation

An OxiSelect™ Protein Carbonyl Immunoblot Kit (Cell Biolabs Inc., San Diego, CA, USA) was used to analyze carbonylated proteins according to the manufacturer’s instructions, with β-actin (Santa Cruz Biotechnology, Dallas, TX, USA) used as an internal loading control.

### 2.7. Preamyloid Oligomer Formation

Preamyloid oligomer formation was assayed in atrial HL-1 myocytes following IsoLG stimulation as described previously [20], with minor modifications. Cells were exposed to IsoLG for various time periods, fixed with 4% paraformaldehyde, and incubated with a conformation-specific, polyclonal rabbit A-11 antibody overnight at 4 °C, followed by incubation with rabbit Alexa Fluor 488 antibody and imaging using confocal microscopy.

### 2.8. Apoptosis

Apoptotic activity in response to IsoLG treatment was monitored using CellEvent™ Caspase-3/7 Green Detection Reagent (Thermo Fisher Scientific, Waltham, MA, USA) according to the manufacturer’s instructions.

### 2.9. Quantitation of Proteins and mRNA

Expression of oxidative phosphorylation proteins in the absence and presence of IsoLG exposure was assayed by Western blotting using a total oxidation phosphorylation (OXPHOS) rodent antibody cocktail (Abcam, Waltham, MA, USA), with β-actin employed as a loading control. Analogous methods were used to quantitate carbonylated proteins. Atrial mRNA expression (e.g., mitochondrial genes) was assayed using qRT-PCR.

### 2.10. Electrophysiology

The whole cell configuration was used to record action potentials and ionic currents from single cells as described previously [14]. Action potentials were recorded from mouse atrial myocytes or atrial HL-1 cells at 37 °C using the current clamp technique, while ionic currents from atrial HL-1 cells (I_Na_ and I_To_), *Ltk*^−^ cells (I_sus_, represented by KCNA5 current), and mouse atrial myocytes (I_Ca,L_) were recorded in voltage clamp experiments at room temperature (22 ± 1 °C).

### 2.11. Statistics

Data are presented as mean ± SEM. Statistical significance was determined by one-way ANOVA and post hoc Tukey tests for multiple comparisons using Graph Pad Prism software (Version 9.1.2). Differences with a *p*-value < 0.05 were considered significant. For electrophysiologic recordings, data were analyzed using Clampfit 10.0 software (Molecular Devices, Sunnyvale, CA, USA), compiled in Excel Office 365 (Microsoft, Redmond, WA, USA), and plotted and fitted in OriginPro 2022 (OriginLab Corporation, Northampton, MA, USA). Unless specified, a Student’s paired *t*-test (two-tailed) was used when appropriate.

## 3. Results

### 3.1. IsoLGs Cause Mitochondrial Dysfunction in Atrial HL-1 Cells

**Figure 1:**
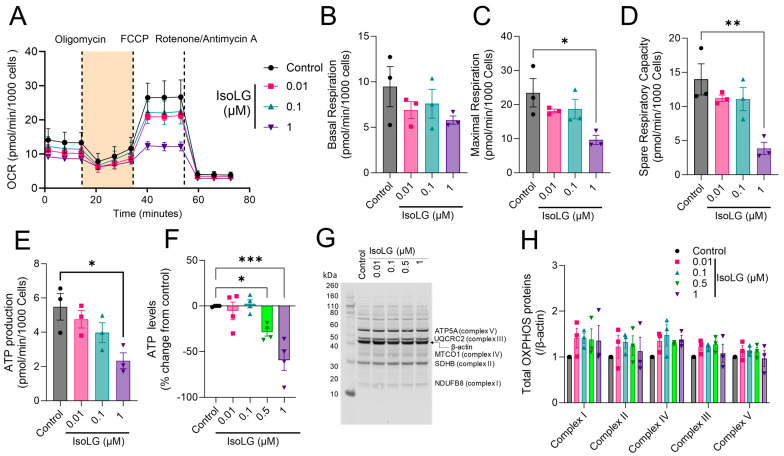
Isolevuglandins (IsoLGs) cause mitochondrial dysfunction in atrial cardiomyocytes. Mitochondrial stress test showing average oxygen consumption rate (OCR) profile (**A**) in atrial HL-1 cells treated with different concentrations of IsoLGs for 2 h compared to control cells. Acute IsoLG exposure decreased mitochondrial respiratory parameters in a concentration-dependent manner, including maximal respiration (**C**), spare respiratory capacity (**D**), and ATP production (**E**), with a trend for basal respiration (**B**). Data represent mean ± SEM from 3 independent experiments; * *p* < 0.05, ** *p* < 0.01 vs. control, one-way ANOVA. All OCR values were normalized to the number of Hoechst positive nuclei. (**F**) Using a luminescence assay, cellular ATP production was reduced following a 2 h exposure to IsoLGs (*n* = 4 independent experiments; * *p* < 0.05, *** *p* <0.001 vs. control, one-way ANOVA). (**G**) Representative Western blot and (**H**) quantitative analysis of total oxidative phosphorylation (OXPHOS) protein expression is displayed in the absence and presence of IsoLG exposure to HL-1 cells (*n* = 3 experiments).

Reactive IsoLGs have been detected in the atria of hypertensive mice with increased AF susceptibility, and they have been implicated in oxidative stress-related mitochondrial dysfunction in hypertensive vasculature [13,21]. To determine whether IsoLGs can alter metabolic function in the atrium, atrial HL-1 cells were exposed to increasing concentrations of IsoLGs for 2 h followed by bioenergetic profiling using the Seahorse XFp Mito Stress Test, with OCR serving as an indicator of mitochondrial function. Compared to control cells, IsoLGs caused a concentration-dependent reduction in maximal respiration, spare respiratory capacity, and ATP production with a similar non-significant trend for basal respiration (Figure 1A–E). In parallel experiments, IsoLG-mediated reduction in cellular ATP production was confirmed using an ATP luminescence assay (Figure 1F). These effects were not associated with any changes in the abundance of representative oxidative phosphorylation proteins in the mitochondrial electron transport chain (ETC) with IsoLG exposure (Figure 1G,H). Collectively, these results demonstrate that IsoLGs have deleterious effects on atrial cardiomyocyte mitochondrial function.

### 3.2. IsoLGs Decrease Mitochondrial Membrane Potential (Δψm) and Promote Apoptosis

**Figure 2:**
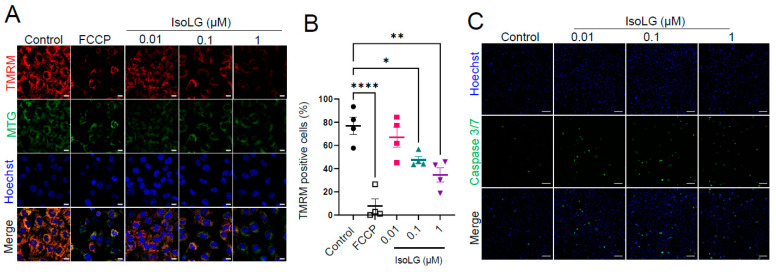
IsoLGs decrease mitochondrial membrane potential and promote apoptosis. (**A**) Representative confocal images of atrial HL-1 cells are shown following a 2 h incubation with different concentrations of IsoLGs, using carbonyl cyanide p-trifluoromethoxyphenylhydrazone (FCCP) as a positive control. Panels represent visualization of mitochondrial membrane potential (tetramethylrhodamine methyl ester [TMRM], top), mitochondrial mass (Mitotracker Green or MTG), and nuclei (Hoechst), with merged images in the bottom panel (scale bar, 10 µm). (**B**) High-throughput content analysis was used to quantify alterations in mitochondrial membrane potential following IsoLG treatment (*n* = 4 experiments; * *p* < 0.05, ** *p* < 0.01, **** *p* < 0.0001 vs. control, one-way ANOVA). (**C**) Confocal images demonstrate apoptotic nuclei (green in the middle panel, using a Caspase 3/7 assay) in HL-1 cells after a 2 h exposure to different concentrations of IsoLGs (scale bar, 100 µm). Hoechst staining was used to visualize cellular DNA/nuclei (blue), with merged images at the bottom.

Given these findings, the effect of IsoLGs on Δψm was assessed using TMRM, a membrane potential-dependent fluorescent probe that sequesters in the matrix of functioning mitochondria [22], and imaging with live-cell confocal microscopy in atrial HL-1 cells. *MitoTracker^®^Green* FM, which accumulates within mitochondria regardless of mitochondrial membrane potential, was used to visualize mitochondria, while FCCP, which uncouples mitochondrial oxidative phosphorylation to collapse Δψm, served as a positive control. IsoLGs caused a concentration-dependent reduction in TMRM fluorescence compared to control cells (Figure 2A,B), indicative of mitochondrial uncoupling and further evidence of mitochondrial dysfunction. In parallel experiments, qualitative assessment demonstrated that IsoLGs also increased atrial myocyte apoptosis (Figure 2C).

### 3.3. IsoLGs Promote Formation of Intracellular Superoxide and Preamyloid Oligomers

**Figure 3:**
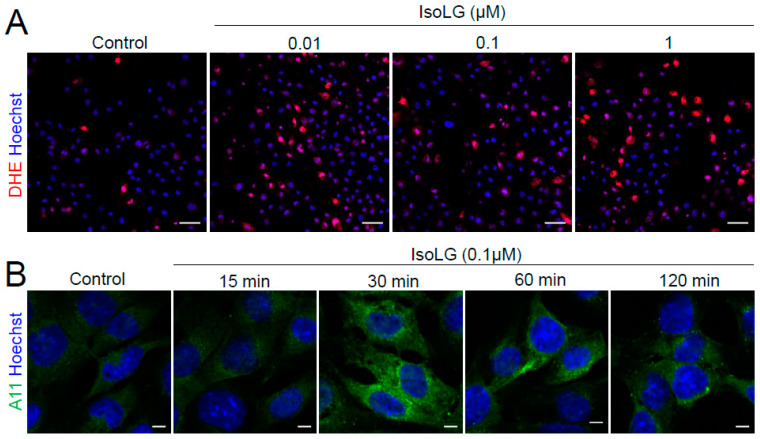
IsoLGs increase intracellular superoxide generation and promote preamyloid oligomer formation. (**A**) Atrial HL-1 cells were pretreated with increasing concentrations of IsoLGs for 2 h, followed by staining with DHE (red) and Hoechst (blue) to visualize superoxide (O_2_^•−^) and nuclei, respectively (scale bar, 50 µm). (**B**) Confocal images demonstrate time-dependent generation of preamyloid oligomers (green) in HL-1 cells exposed to IsoLGs (scale bar, 5 µm).

Mitochondria are a major site of ROS generation in cardiomyocytes [23]. To determine whether IsoLGs could alter the cellular redox status, atrial cells were incubated with increasing concentrations of IsoLGs, followed by exposure to DHE to visualize cytosolic O_2_^•−^ during live cell confocal microscopy. Interestingly, ROS generation was increased in IsoLG-treated cells compared to the control cells (Figure 3A), indicating a feed-forward effect by these mediators. In atrial HL-1 cells, we previously demonstrated that IsoLGs accelerate protein misfolding of natriuretic peptides to cause the formation of preamyloid oligomers that produce pro-arrhythmic electrophysiologic and metabolic effects [11,14,20]. Experiments were performed to determine the time course of this effect. As illustrated in Figure 3B, there was no effect of IsoLG treatment at 15 min of exposure, indicating that, as expected, oligomer formation was not immediate, but rather a discernible increase in oligomers was evident within 30 min. For untreated control cells, there was minimal development of preamyloid oligomers over 120 min (Figure S1 in the Supplementary Materials).

### 3.4. 2-HOBA Blunts IsoLG-Mediated Mitochondrial Dysfunction

**Figure 4:**
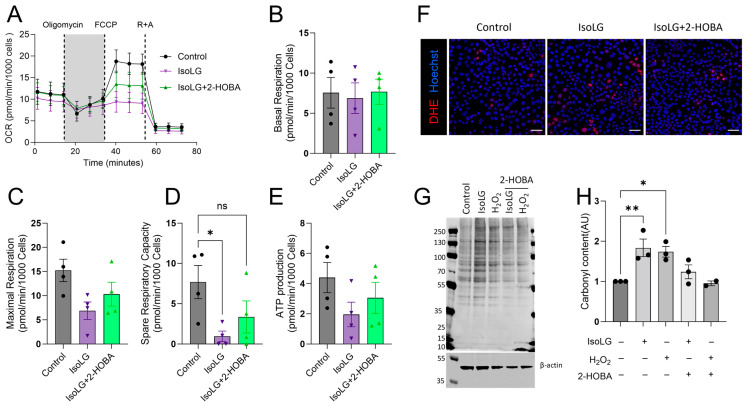
2-HOBA effects on IsoLG-mediated mitochondrial dysfunction, O_2_^•−^ formation, and protein carbonyl content. (**A**) Atrial HL-1 cells were preincubated for 24 h with 2-HOBA (100 µm) or vehicle prior to IsoLG (1 µm) treatment for 2 h. (**A**) Average OCR profiles are illustrated in control cells, and cells treated with IsoLGs in the presence or absence of 2-HOBA. Mitochondrial respiratory parameters include basal respiration (**B**), maximal respiration (**C**), spare respiratory capacity (**D**), and ATP production (**E**). Data represent mean ± SEM from 4 independent experiments. All OCR values were normalized to the number of Hoechst positive nuclei. (**F**) Cells were pretreated with 2-HOBA (100 µm) or vehicle for 24 h prior to IsoLG (1 µm) exposure for 2 h in the absence or presence of 2-HOBA. Cells were stained with DHE to detect O_2_^•−^, followed by live cell imaging (scale bar, 50 µm). 2-HOBA prevented the IsoLG-induced increase in intracellular O_2_^•−^. (**G**) In parallel experiments, cells were cultured for an additional 24 h in Claycomb media after IsoLG treatment followed by harvest, with cell lysates subjected to Western blot analysis to detect protein carbonylation, using H_2_O_2_ (50 µm) as a positive control. (**H**) Quantification was performed for protein levels in panel (**G**) (*n* = 3 experiments; * *p* < 0.05, ** *p* < 0.01 vs. control, one-way ANOVA followed by Tukey’s post hoc analysis).

To investigate whether IsoLG-induced mitochondrial dysfunction could be prevented by the dicarbonyl scavenger 2-HOBA, atrial HL-1 cells were incubated with 2-HOBA or vehicle prior to IsoLG exposure, followed by an assessment of mitochondrial oxidative function, ROS generation, and protein peroxidation. Consistent with our previous findings, IsoLGs tended to suppress basal and maximal respiration, spare respiratory capacity, and ATP production compared to control cells, although these changes were only statistically significant for spare respiratory capacity (Figure 4A–E). In addition, there was a largely nonsignificant trend for 2-HOBA pretreatment to blunt these deleterious effects on mitochondrial bioenergetics. Similarly, 2-HOBA reduced IsoLG-mediated ROS generation in atrial cells (Figure 4F). Protein carbonylation is an additional indicator of ROS-mediated oxidative damage that can cause cellular dysfunction [24,25,26]. As expected, protein carbonyl content was increased by exposure to IsoLG or H_2_O_2_ (positive control; Figure 4G,H), but this effect was blunted by 2-HOBA. Thus, 2-HOBA tended to reduce ROS generation to protect against IsoLG-induced mitochondrial dysfunction and protein modification, suggesting that the effects of IsoLGs were largely mediated by lipid dicarbonyls.

### 3.5. 2-HOBA Protects against IsoLG-Induced Mitochondrial ROS, as well as Disruption of Mitochondrial Network and Content

**Figure 5:**
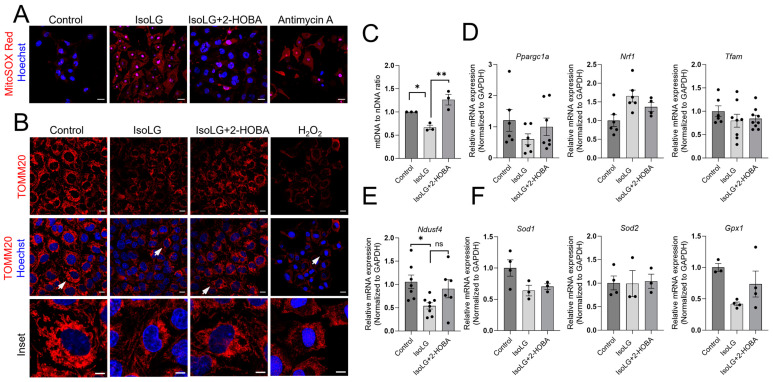
2-Hydroxybenzylamine (2-HOBA) protects against IsoLG-induced mitochondrial O_2_^•−^ generation and disruption of mitochondrial network and content. Atrial HL-1 cells were pretreated with 2-HOBA (100 µm) or vehicle (control) for 24 h prior to IsoLG (1µm) exposure for 2 h in the absence or presence of 2-HOBA. Cells were then stained with MitoSOX Red ((**A**), antimycin A was a positive control; scale bar, 20 µm) or, following an additional 24 h of culture in Claycomb media, with TOMM20 ((**B**), H_2_O_2_ was a positive control; scale bar, 10 µm). In (**B**), the arrows in the middle panels point to cells highlighted in the inset images shown in the lower panel. In parallel experiments, the cells were harvested 2 h after IsoLG treatment in the absence or presence of 2-HOBA, with mitochondrial DNA (mtDNA) or total RNA extracted and subjected to qPCR or qRT-PCR to determine mtDNA content ((**C**), * *p* < 0.05, ** *p* < 0.01 vs. control, one-way ANOVA followed by Tukey’s post hoc analysis), expression of *Ndusf4* mRNA ((**E**), * *p* < 0.05, ns is nonsignificant vs. control, one-way ANOVA followed by Tukey’s post hoc analysis), or expression of multiple genes related to mitochondrial biogenesis or antioxidant capacity (**D**,**F**).

To investigate whether IsoLGs increase the production of ROS specifically in the mitochondria, atrial myocytes were exposed to IsoLGs in the absence or presence of 2-HOBA followed by staining with MitoSOX Red and confocal imaging. Antimycin A, a complex III inhibitor, was used as a positive control. As illustrated in Figure 5A, cells treated with IsoLGs alone displayed a marked increase in mitochondrial ROS generation. However, this effect was significantly blunted by pretreatment with 2-HOBA, indicating an effect to scavenge mitochondrial IsoLGs.

To further investigate the protective effects of 2-HOBA on IsoLG-induced mitochondrial dysfunction, we evaluated common biomarkers of mitochondrial network and content, as well as mtDNA. Following preincubation in the absence or presence of 2-HOBA, atrial HL-1 cells were exposed to IsoLGs and immunostained with the mitochondria structural marker TOMM20, with H_2_O_2_ as a positive control. By confocal microscopy, control cells demonstrated normal mitochondria content and distribution, characterized by an evenly distributed network radiating from the nucleus (Figure 5B). In IsoLG-treated cells, mitochondria number and network structure were diminished compared to control cells, and this damage was reduced by pretreatment with 2-HOBA. As expected, cells treated with H_2_O_2_ demonstrated nearly complete loss of mitochondrial staining indicative of organelle loss, with smaller and more brightly staining nuclei, reflecting apoptosis.

Mitochondrial DNA damage caused by oxidative stress is implicated in the pathophysiology of various cardiovascular diseases including atrial fibrillation and heart failure [27,28,29,30]. To investigate whether IsoLGs can cause this abnormality, mtDNA was quantified using qPCR. Figure 5C demonstrates a reduction in mtDNA in IsoLG-treated cells, and this was prevented by pretreatment with 2-HOBA. Finally, IsoLG exposure caused a significant decrease in the mRNA expression of Ndusf4, a component of Complex I of the mitochondrial electron transport chain (Figure 5E), corroborating our previous findings regarding mitochondrial function. There was a trending but nonsignificant effect of 2-HOBA in preventing this effect. On the other hand, there were no significant effects of IsoLGs on the expression of other genes associated with mitochondrial biogenesis and antioxidant capacity (Figure 5D,F). Collectively, these results indicate that 2-HOBA protects against IsoLG-mediated mitochondrial structural dysfunction.

### 3.6. IsoLGs Promote Pro-Arrhythmic Electrophysiologic Effects in Murine Atrial Myocytes and Atrial HL-1 Cells

**Figure 6:**
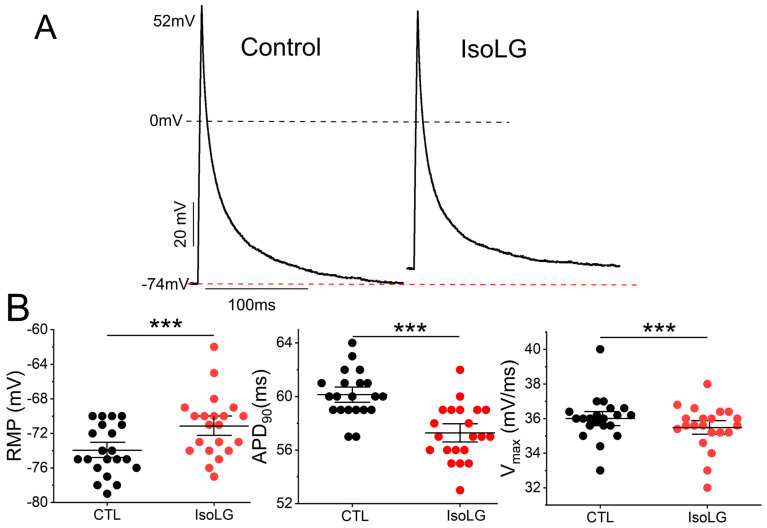
IsoLGs modulate atrial action potentials in a pro-arrhythmic manner. (**A**) Representative examples of mouse atrial action potentials stimulated at 1 Hz are illustrated before (Control) and following bath exposure to IsoLG 500 nM. The red dashed line is the resting membrane potential under control conditions and the black dashed line represents 0 mV. Control values are shown for resting membrane potential (RMP) and the action potential (AP) overshoot. AP amplitude was 126 and 124 mV for control and IsoLG APs, respectively. (**B**) Summary data demonstrate that IsoLG exposure causes abbreviation of action potential duration at 90% repolarization (APD_90_; center) and a reduction in the maximum rate of rise of phase 0 (V_max_; right) compared to control (CTL), as well as elevation of the resting membrane potential (RMP, left); *n* = 21; *** *p* < 0.001, Wilcoxon signed-rank test.

Previous studies have demonstrated that IsoLGs can modulate at least some cardiac ionic currents, including the voltage-gated Na^+^ current as well as the K^+^ current from rapidly activating delayed rectifier channels I_Kr_ (absent in the mouse atrium) [10]. Accordingly, we investigated whether IsoLGs could modify action potentials (APs) in single mouse atrial myocytes. As illustrated by representative examples in Figure 6A and summary data in Figure 6B and Table S1, IsoLGs caused a reduction in both AP duration (APD at 90% repolarization, or APD_90_) and the maximum rate of rise of phase 0 (V_max_), as well as elevation of the resting membrane potential (RMP). Moreover, these effects were rapid, beginning immediately upon bath exposure and typically reaching a steady state within 10–15 min. Such changes are predicted to be pro-arrhythmic, as shortened repolarization and slowed conduction promote the development of re-entrant circuits. Similar results were seen in atrial HL-1 cells (Table S2). These deleterious effects on atrial action potentials were prevented when cells were initially preincubated in 2-HOBA prior to IsoLG exposure (Figure S2, Tables S3 and S4).

### 3.7. IsoLGs Reduce Inward Na^+^ and Ca^2+^ Currents and Increase Outward K^+^ Currents

**Figure 7:**
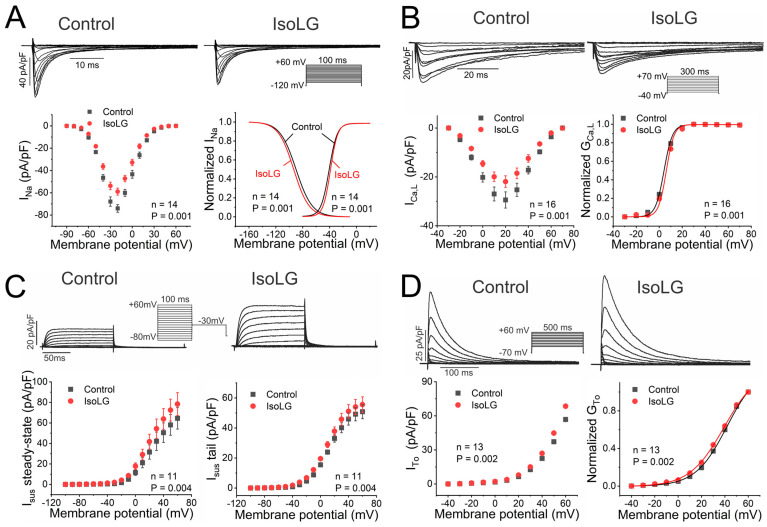
Amplitude and gating are altered for multiple ionic currents with IsoLG exposure. (**A**) Representative families of Na^+^ currents are illustrated before and after bath application of IsoLG 500 nM using the voltage clamp protocol shown in the inset (**top**). Summary data are illustrated for the peak current-voltage relationship (**left**) and curves representing the voltage dependence of normalized channel inactivation (**left**) and the normalized conductance (G)-voltage plot, or voltage dependence of activation (**right**) in the absence (black) and presence (red) of IsoLG exposure (*p* values vs. control; n is shown on the Figure; Wilcoxon signed-rank test for peak current amplitude and curve midpoints [V_1/2_]). Similar data are shown for L-type Ca^2+^ current (**B**), with associated current-voltage relationship and activation curves; sustained outward K^+^ current I_sus_ (**C**) with current-voltage relationships for steady-state and tail currents; and the transient outward K^+^ current I_To_ (**D**) with its current-voltage relationship and activation curves.

Voltage clamp experiments were performed to investigate the mechanistic basis of the observed IsoLG-mediated AP changes. As reported previously [31], IsoLG exposure suppressed cardiac Na^+^ current, with a shift in channel inactivation to more negative potentials (Figure 7A, Table S6). In addition, we observed a small depolarizing shift in channel activation that was not previously noted (Figure 7A; Table S5). Similarly, IsoLGs caused a reduction in the L-type Ca^2+^ current, with a rightward shift in channel activation (Figure 7B, Tables S5 and S6). On the other hand, both I_sus_ and the transient outward K^+^ current were increased in response to IsoLG exposure, with negative shifts in channel activation (Figure 7C,D, Tables S5 and S6). As observed for AP changes, these effects occurred rapidly upon IsoLG exposure with a similar time course. A reduced Na^+^ current would explain the decrease in V_max_, while a reduction in I_Ca,L_, as well as increases in I_sus_ and I_To_, would shorten APD to account for these effects on the cardiac AP.

## 4. Discussion

In this study, we have identified IsoLGs as novel effectors of oxidative stress-mediated atrial cell dysfunction, characterized by mitochondrial and electrophysiologic alterations that are potentially pro-arrhythmic. In addition, our results demonstrate two distinct mechanisms by which these detrimental effects occur. First, IsoLGs interact with and directly modify cardiomyocyte proteins, best illustrated by their rapid effects on ion channel function at the plasma membrane. Second, IsoLGs promote the generation of cytotoxic protein oligomers, which themselves have also been shown to cause electrophysiologic and bioenergetic dysfunction in atrial cardiomyocytes [14]. Interestingly, we also found that IsoLGs promote additional cellular ROS production, leading to a detrimental feed-forward effect. Importantly, the pro-arrhythmic effects of IsoLGs on atrial cardiomyocytes were ameliorated by pretreatment with 2-HOBA, an IsoLG scavenger. Taken together, our data indicate that targeting IsoLGs to reduce the generation of IsoLG-protein adducts in the atria represents a novel therapeutic approach to the prevention of AF.

Multiple lines of evidence have implicated inflammation and excessive ROS production in AF risk factors such as hypertension, as well as the pathogenesis of AF itself [11,32,33,34,35,36,37]. However, results from experimental and clinical studies using antioxidant therapies to prevent AF have been disappointing [38], possibly due to ineffective dosing, or the impairment of host defenses and/or disruption of physiologic ROS signaling. Emerging evidence indicates that ROS-mediated lipid peroxidation, resulting in the formation of dicarbonyl compounds such as IsoLGs, plays an important role in the pathogenesis of many human diseases, including cardiovascular disorders [10]. IsoLGs are the most reactive lipid dicarbonyl products identified to date, and they rapidly adduct to primary amines, including lysyl residues of proteins, to form irreversible covalent modifications [39,40]. Preclinical studies using IsoLG scavengers such as 2-HOBA have demonstrated benefits in atherosclerosis [12], hypertension [11], and heart failure [41]. Moreover, we recently found that 2-HOBA prevented electrical remodeling and AF susceptibility in a murine model of hypertension [11,14], suggesting that IsoLGs along with preamyloid oligomers likely participate in pro-arrhythmic, hypertension-mediated atrial remodeling. Importantly, scavengers of these mediators differ mechanistically from contemporary antioxidants, as they do not target ROS generation and therefore are more likely to preserve physiologic ROS signaling.

It is well-recognized that electrophysiologic remodeling can initiate and/or perpetuate AF. In mouse atrial myocytes, IsoLGs caused a reduction in APD and V_max_, both of which would promote re-entry. In our electrophysiologic experiments, modulation of action potentials and ionic currents developed rapidly upon IsoLG exposure, typically reaching a steady state within 15 min, supporting a direct effect on cardiac ion channel proteins. This is consistent with previous studies exploring the role of the Na_v_1.5 channel in ischemic ventricular arrhythmias arising from the canine myocardial infarct border zone, where IsoLGs accumulate [31]. Using heterologously expressed human Na_v_1.5 channels and atrial HL-1 cells, the oxidant tert-butyl-hydroperoxide or IsoLG caused a reduction in Na^+^ current accompanied by a leftward shift in channel availability, and these effects were prevented by 2-HOBA. Using click chemistry, it was demonstrated that these changes were associated with direct lipoxidative modification of the Na^+^ channel protein [42]. In addition, IsoLGs have also been shown to oxidatively modify human HDL cholesterol to cause dysfunction, while 2-HOBA has been shown to attenuate atherosclerosis in murine models [12].

In addition to electrophysiologic derangements, there is increasing evidence that mitochondrial dysfunction participates in the pathophysiology of AF. Atrial tissue from patients with AF demonstrates impaired mitochondrial respiration, mtDNA deletions, disturbances in the atrial transcriptome for OXPHOS-related gene expression, and abundant evidence of increased ROS [5,6,33,43]. Subcellular anatomy is critical for optimal mitochondrial function, as the close physical relationship of the mitochondria and SR is essential for effective mitochondrial Ca^2+^ buffering. Interestingly, the atria of AF patients have displayed disruption of this mitochondrial network [4,6]. Our results in atrial cardiomyocytes indicate that IsoLGs impaired mitochondrial respiration, reduced mitochondrial membrane potential, and disturbed the mitochondrial network structure. These results corroborate previous findings that IsoLGs alter mitochondrial respiration and membrane potential [44]. In addition, IsoLGs increased cytoplasmic and mitochondrial oxidative stress, findings consistent with the concept of a feed-forward generation mechanism by IsoLGs [45]. A potential explanation for this phenomenon is that the collapse of Δψm would shunt NADH/NADPH towards restoration of this gradient at the expense of ROS scavenging, leading to an increase in mitochondrial ROS production [9]. Our data are also consistent with a previous report demonstrating that acute IsoLG exposure inhibits mitochondrial respiration and attenuates Complex I activity in isolated murine kidney mitochondria [21]. Moreover, we found that IsoLG treatment decreased expression of the complex I subunit *Ndufs4* in atrial cardiomyocytes, which was prevented by 2-HOBA. Complex I is the largest of the mitochondrial ETC complexes and a major source of ROS. Previously, it was shown that cardiac-specific *Ndufs4*-null mice developed cardiomyopathy with a significant decrease in cardiac complex I activity, while *Ndufs4* knockout also demonstrated reduced complex I- driven oxygen consumption [46], indicating a vital role of *Ndufs4* in maintaining complex I activity.

While IsoLGs promote multiple effects reflecting mitochondrial dysfunction in atrial cardiomyocytes, we provide the first experimental evidence indicating they can promote a reduction in mtDNA and mitochondrial mass, characterized by a decrease in the mtDNA to nuclear DNA ratio and reduced TOMM20 staining. Prior studies have implicated the presence of mtDNA damage due to excessive ROS generation in other cardiac pathologies, including ischemia-reperfusion injury and heart failure, as well as AF [47,48,49,50]. Notably, oxidative injury-induced cardiac mtDNA damage was increased in the atria of patients with AF [30], while mitochondria DNA copy number was inversely associated with the risk of AF in human subjects [51]. In our model, it is likely that the increased mitochondrial ROS generated by IsoLGs contributes to the reduction of mtDNA content.

Because natriuretic peptide-derived atrial amyloid and a mutation in *NPPA* encoding atrial natriuretic peptide (ANP) are both associated with AF, we previously investigated the effects of natriuretic peptide preamyloid oligomers on atrial cell function. Interestingly, oligomer-mediated electrophysiologic and metabolic effects were reminiscent of those of IsoLGs, and IsoLGs have been shown to accelerate oligomer formation. Thus, it is likely that indirect effects of oligomer production account for at least some of the detrimental effects of IsoLGs, in particular metabolic effects. However, given that it takes at least 30 min for oligomers to form with IsoLG exposure, they cannot mediate the rapid effects on ion channel function that we observed.

The limitation of this investigation is the use of atrial HL-1 cells for the bioenergetic studies that were performed. However, it is well-recognized that collagenase digestion causes cardiomyocyte injury leading to decreased viability over time, especially for atrial myocytes. Based on this consideration, we and other investigators have utilized atrial HL-1 cells for such studies based on their preserved bioenergetic capacity [52].

In conclusion, our findings indicate that highly reactive IsoLGs alter atrial cell electrophysiology and mitochondrial function in a pro-arrhythmic manner, thus representing a significant component of oxidative stress-mediated atrial cell injury. Pharmacologic efforts to target these and other lipid dicarbonyls represent a novel and promising therapeutic approach to prevent detrimental atrial remodeling and AF susceptibility.

## Data Availability

The data presented in this study are available in the article text or Supplementary Materials.

## Supplementary Materials

The following supporting information can be downloaded at: https://www.mdpi.com/article/10.3390/cells13060483/s1. References [11,14,15,16,17,18,19,20,53] are cited in the supplementary materials.

## References

[1] Andrade J., Khairy P., Dobrev D., Nattel S. (2014). The clinical profile and pathophysiology of atrial fibrillation: Relationships among clinical features, epidemiology, and mechanisms. Circ. Res..

[2] Griendling K.K., Camargo L.L., Rios F.J., Alves-Lopes R., Montezano A.C., Touyz R.M. (2021). Oxidative Stress and Hypertension. Circ. Res..

[3] Yang K.C., Bonini M.G., Dudley S.C. (2014). Mitochondria and arrhythmias. Free. Radic. Biol. Med..

[4] Wiersma M., van Marion D.M.S., Wust R.C.I., Houtkooper R.H., Zhang D., Groot N.M.S., Henning R.H., Brundel B. (2019). Mitochondrial Dysfunction Underlies Cardiomyocyte Remodeling in Experimental and Clinical Atrial Fibrillation. Cells.

[5] Emelyanova L., Ashary Z., Cosic M., Negmadjanov U., Ross G., Rizvi F., Olet S., Kress D., Sra J., Tajik A.J. (2016). Selective downregulation of mitochondrial electron transport chain activity and increased oxidative stress in human atrial fibrillation. Am. J. Physiol. Heart Circ. Physiol..

[6] Bukowska A., Schild L., Keilhoff G., Hirte D., Neumann M., Gardemann A., Neumann K.H., Rohl F.W., Huth C., Goette A. (2008). Mitochondrial dysfunction and redox signaling in atrial tachyarrhythmia. Exp. Biol. Med..

[7] Avula U.M.R., Dridi H., Chen B.X., Yuan Q., Katchman A.N., Reiken S.R., Desai A.D., Parsons S., Baksh H., Ma E. (2021). Attenuating persistent sodium current-induced atrial myopathy and fibrillation by preventing mitochondrial oxidative stress. JCI Insight.

[8] Liu C., Bai J., Dan Q., Yang X., Lin K., Fu Z., Lu X., Xie X., Liu J., Fan L. (2021). Mitochondrial Dysfunction Contributes to Aging-Related Atrial Fibrillation. Oxidative Med. Cell. Longev..

[9] Mason F.E., Pronto J.R.D., Alhussini K., Maack C., Voigt N. (2020). Cellular and mitochondrial mechanisms of atrial fibrillation. Basic Res. Cardiol..

[10] May-Zhang L.S., Kirabo A., Huang J., Linton M.F., Davies S.S., Murray K.T. (2021). Scavenging Reactive Lipids to Prevent Oxidative Injury. Annu. Rev. Pharmacol. Toxicol..

[11] Prinsen J.K., Kannankeril P.J., Sidorova T.N., Yermalitskaya L.V., Boutaud O., Zagol-Ikapitte I., Barnett J.V., Murphy M.B., Subati T., Stark J.M. (2020). Highly-reactive isolevuglandins promote atrial fibrillation caused by hypertension. JACC Basic Transl. Sci..

[12] Tao H., Huang J., Yancey P.G., Yermalitsky V., Blakemore J.L., Zhang Y., Ding L., Zagol-Ikapitte I., Ye F., Amarnath V. (2020). Scavenging of reactive dicarbonyls with 2-hydroxybenzylamine reduces atherosclerosis in hypercholesterolemic Ldlr(−/−) mice. Nat. Commun..

[13] Dikalova A., Mayorov V., Xiao L., Panov A., Amarnath V., Zagol-Ikapitte I., Vergeade A., Ao M., Yermalitsky V., Nazarewicz R.R. (2020). Mitochondrial Isolevuglandins Contribute to Vascular Oxidative Stress and Mitochondria-Targeted Scavenger of Isolevuglandins Reduces Mitochondrial Dysfunction and Hypertension. Hypertension.

[14] Yang Z., Subati T., Kim K., Murphy M.B., Dougherty O.P., Christopher I.L., Van Amburg J.C., Woodall K.K., Barnett J.V., Murray K.T. (2022). Natriuretic Peptide Oligomers Cause Proarrhythmic Metabolic and Electrophysiological Effects in Atrial Myocytes. Circ Arrhythm Electrophysiol.

[15] Claycomb W.C., Lanson N.A., Stallworth B.S., Egeland D.B., Delcarpio J.B., Bahinski A., Izzo N.J. (1998). HL-1 cells: A cardiac muscle cell line that contracts and retains phenotypic characteristics of the adult cardiomyocyte. Proc. Natl. Acad. Sci. USA.

[16] Yang Z., Shen W., Rottman J.N., Wikswo J.P., Murray K.T. (2005). Rapid stimulation causes electrical remodeling in cultured atrial myocytes. J. Mol. Cell. Cardiol..

[17] Miller S.A., Dykes D.D., Polesky H.F. (1988). A simple salting out procedure for extracting DNA from human nucleated cells. Nucleic Acids Res..

[18] Malik A.N., Czajka A., Cunningham P. (2016). Accurate quantification of mouse mitochondrial DNA without co-amplification of nuclear mitochondrial insertion sequences. Mitochondrion.

[19] Livak K.J., Schmittgen T.D. (2001). Analysis of relative gene expression data using real-time quantitative PCR and the 2(-Delta Delta C(T)) Method. Methods.

[20] Sidorova T.N., Yermalitskaya L.V., Mace L.C., Wells K.S., Boutaud O., Prinsen J.K., Davies S.S., Roberts L.J., Dikalov S.I., Glabe C.G. (2015). Reactive γ-ketoaldehydes promote protein misfolding and preamyloid oligomer formation in rapidly-activated atrial cells. J. Mol. Cell. Cardiol..

[21] Mayorov V., Uchakin P., Amarnath V., Panov A.V., Bridges C.C., Uzhachenko R., Zackert B., Moore C.S., Davies S., Dikalova A. (2019). Targeting of reactive isolevuglandins in mitochondrial dysfunction and inflammation. Redox Biol..

[22] Chazotte B. (2011). Labeling mitochondria with TMRM or TMRE. Cold Spring Harb. Protoc..

[23] Bou-Teen D., Kaludercic N., Weissman D., Turan B., Maack C., Di Lisa F., Ruiz-Meana M. (2021). Mitochondrial ROS and mitochondria-targeted antioxidants in the aged heart. Free Radic. Biol. Med..

[24] Nabeshi H., Oikawa S., Inoue S., Nishino K., Kawanishi S. (2006). Proteomic analysis for protein carbonyl as an indicator of oxidative damage in senescence-accelerated mice. Free Radic. Res..

[25] Chevion M., Berenshtein E., Stadtman E.R. (2000). Human studies related to protein oxidation: Protein carbonyl content as a marker of damage. Free Radic. Res..

[26] Aldini G., Dalle-Donne I., Colombo R., Maffei Facino R., Milzani A., Carini M. (2006). Lipoxidation-derived reactive carbonyl species as potential drug targets in preventing protein carbonylation and related cellular dysfunction. ChemMedChem.

[27] Barja G., Herrero A. (2000). Oxidative damage to mitochondrial DNA is inversely related to maximum life span in the heart and brain of mammals. FASEB J..

[28] Tsutsui H., Kinugawa S., Matsushima S. (2008). Oxidative stress and mitochondrial DNA damage in heart failure. Circ. J..

[29] Pool L., Wijdeveld L., de Groot N.M.S., Brundel B. (2021). The Role of Mitochondrial Dysfunction in Atrial Fibrillation: Translation to Druggable Target and Biomarker Discovery. Int. J. Mol. Sci..

[30] Lin P.H., Lee S.H., Su C.P., Wei Y.H. (2003). Oxidative damage to mitochondrial DNA in atrial muscle of patients with atrial fibrillation. Free Radic. Biol. Med..

[31] Fukuda K., Davies S.S., Nakajima T., Ong B.H., Kupershmidt S., Fessel J., Amarnath V., Anderson M.E., Boyden P.A., Viswanathan P.C. (2005). Oxidative mediated lipid peroxidation recapitulates proarrhythmic effects on cardiac sodium channels. Circ. Res..

[32] Muszynski P., Bonda T.A. (2021). Mitochondrial Dysfunction in Atrial Fibrillation-Mechanisms and Pharmacological Interventions. J. Clin. Med..

[33] Montaigne D., Marechal X., Lefebvre P., Modine T., Fayad G., Dehondt H., Hurt C., Coisne A., Koussa M., Remy-Jouet I. (2013). Mitochondrial dysfunction as an arrhythmogenic substrate: A translational proof-of-concept study in patients with metabolic syndrome in whom post-operative atrial fibrillation develops. J. Am. Coll. Cardiol..

[34] Liang X., Zhang Q., Wang X., Yuan M., Zhang Y., Xu Z., Li G., Liu T. (2018). Reactive oxygen species mediated oxidative stress links diabetes and atrial fibrillation. Mol. Med. Rep..

[35] Sovari A.A., Dudley S.C. (2012). Reactive oxygen species-targeted therapeutic interventions for atrial fibrillation. Front. Physiol..

[36] Yang X., An N., Zhong C., Guan M., Jiang Y., Li X., Zhang H., Wang L., Ruan Y., Gao Y. (2020). Enhanced cardiomyocyte reactive oxygen species signaling promotes ibrutinib-induced atrial fibrillation. Redox Biol..

[37] Brookes P.S., Levonen A.L., Shiva S., Sarti P., Darley-Usmar V.M. (2002). Mitochondria: Regulators of signal transduction by reactive oxygen and nitrogen species. Free Radic. Biol. Med..

[38] Violi F., Pastori D., Pignatelli P., Loffredo L. (2014). Antioxidants for prevention of atrial fibrillation: A potentially useful future therapeutic approach? A review of the literature and meta-analysis. Europace.

[39] Davies S.S., May-Zhang L.S. (2018). Isolevuglandins and cardiovascular disease. Prostaglandins Other Lipid Mediat..

[40] Davies S.S., May-Zhang L.S., Boutaud O., Amarnath V., Kirabo A., Harrison D.G. (2020). Isolevuglandins as mediators of disease and the development of dicarbonyl scavengers as pharmaceutical interventions. Pharmacol. Ther..

[41] Shang L., Weng X., Wang D., Yue W., Mernaugh R., Amarnath V., Weir E.K., Dudley S.C., Xu Y., Hou M. (2019). Isolevuglandin scavenger attenuates pressure overload-induced cardiac oxidative stress, cardiac hypertrophy, heart failure and lung remodeling. Free Radic. Biol. Med..

[42] Nakajima T., Davies S.S., Matafonova E., Potet F., Amarnath V., Tallman K.A., Serwa R.A., Porter N.A., Balser J.R., Kupershmidt S. (2010). Selective γ-ketoaldehyde scavengers protect Nav1.5 from oxidant-induced inactivation. J. Mol. Cell. Cardiol..

[43] Jeganathan J., Saraf R., Mahmood F., Pal A., Bhasin M.K., Huang T., Mittel A., Knio Z., Simons R., Khabbaz K. (2017). Mitochondrial Dysfunction in Atrial Tissue of Patients Developing Postoperative Atrial Fibrillation. Ann. Thorac. Surg..

[44] Stavrovskaya I.G., Baranov S.V., Guo X., Davies S.S., Roberts L.J., Kristal B.S. (2010). Reactive ketoaldehydes formed via the isoprostane pathway disrupt mitochondrial respiration and calcium homeostasis. Free Radic. Biol. Med..

[45] Mayorov V., Panov A., Roberts L.J., Vergeade A., Fessel J., Dikalov S. (2014). Feed-Forward Regulation of Oxidative Stress by Isoketal-Mediated Inactivation of Mitochondrial Superoxide Dismutase and Complex, I. Free. Radic. Biol. Med..

[46] Kruse S.E., Watt W.C., Marcinek D.J., Kapur R.P., Schenkman K.A., Palmiter R.D. (2008). Mice with mitochondrial complex I deficiency develop a fatal encephalomyopathy. Cell Metab..

[47] Suematsu N., Tsutsui H., Wen J., Kang D., Ikeuchi M., Ide T., Hayashidani S., Shiomi T., Kubota T., Hamasaki N. (2003). Oxidative stress mediates tumor necrosis factor-alpha-induced mitochondrial DNA damage and dysfunction in cardiac myocytes. Circulation.

[48] Corral-Debrinski M., Stepien G., Shoffner J.M., Lott M.T., Kanter K., Wallace D.C. (1991). Hypoxemia is associated with mitochondrial DNA damage and gene induction. Implications for cardiac disease. JAMA.

[49] Bliksoen M., Baysa A., Eide L., Bjoras M., Suganthan R., Vaage J., Stenslokken K.O., Valen G. (2015). Mitochondrial DNA damage and repair during ischemia-reperfusion injury of the heart. J. Mol. Cell. Cardiol..

[50] Tsutsui H., Ide T., Kinugawa S. (2006). Mitochondrial oxidative stress, DNA damage, and heart failure. Antioxid. Redox Signal..

[51] Zhao D., Bartz T.M., Sotoodehnia N., Post W.S., Heckbert S.R., Alonso A., Longchamps R.J., Castellani C.A., Hong Y.S., Rotter J.I. (2020). Mitochondrial DNA copy number and incident atrial fibrillation. BMC Med..

[52] Antzelevitch C., Brugada P., Borggrefe M., Brugada J., Brugada R., Corrado D., Gussak I., LeMarec H., Nademanee K., Perez Riera A.R. (2005). Brugada syndrome: Report of the second consensus conference. Heart Rhythm.

[53] Amarnath V., Amarnath K., Masterson T.S., Davies S., Roberts L.J. (2005). A simplified synthesis of the diasteromers of levuglandin E2. Synth. Commun..

