# Supplementary material for: Isolevuglandins Promote Mitochondrial Dysfunction and Electrophysiologic Abnormalities in Atrial Cardiomyocytes
Source: Cells. 2024 Mar 9;13(6):483. doi: 10.3390/cells13060483 (PMC10969716; doi:10.3390/cells13060483)
Supplement: Supplementary file 1 [file cells-13-00483-s001.zip › cells-2801982-supplementary.pdf]

SUPPLEMENTARY MATERIALS

Isolevuglandins promote mitochondrial dysfunction and electrophysiologic abnormalities in atrial cardiomyocytes

Tuerdi Subati, Zhenjiang Yang, Matthew B. Murphy, Joshua M. Stark, David Z. Trykall, Sean S. Davies, Joey V. Barnett and Katherine T. Murray

Departments of Medicine and Pharmacology, Vanderbilt University School of Medicine, Nashville, TN

Address for correspondence and reprints:
Dr. Katherine T. Murray
Division of Clinical Pharmacology
Room 559 Preston Research Building
Vanderbilt University School of Medicine
2220 Pierce Avenue
Nashville, TN 37232-6602
Phone: (615) 936-3420
Fax: (615) 936-2746
Email: kathy.murray@vanderbilt.edu

I. SUPPLEMENTARY FIGURES

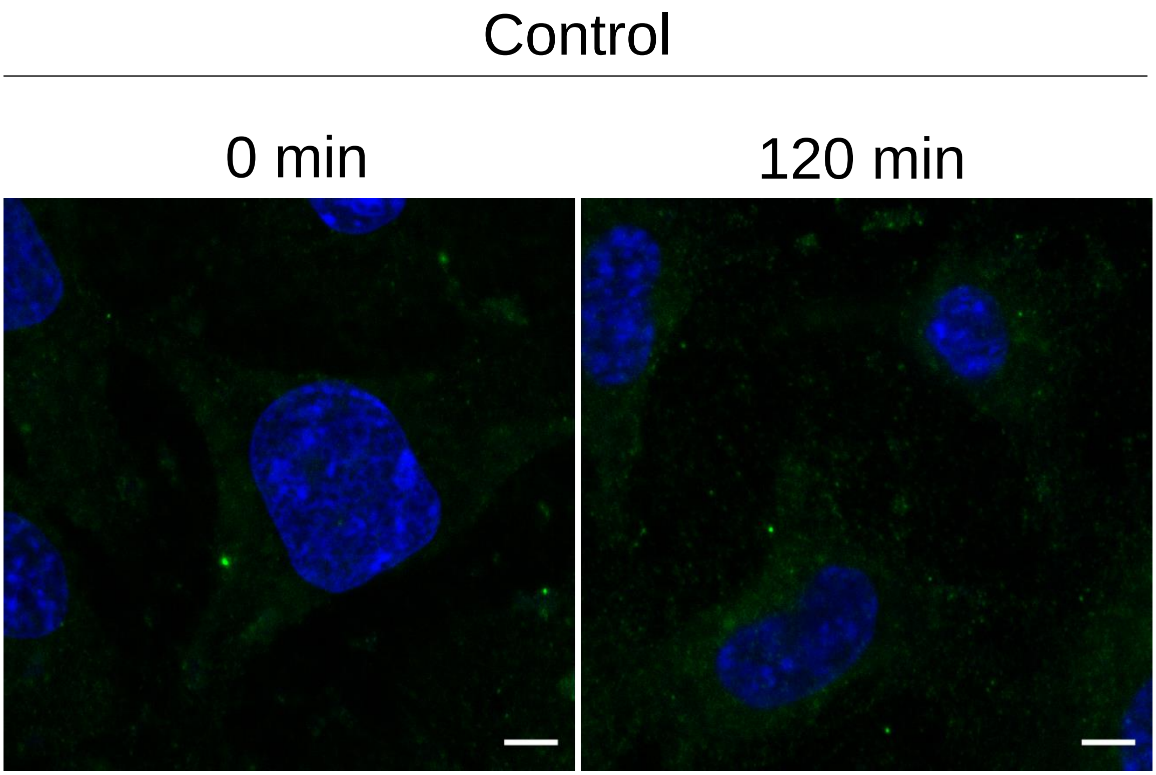

Figure S1: Lack of preamyloid oligomer formation in atrial HL-1 cells under control conditions. Cells were stained with A-11 (green) and Hoechst (blue) to visualize preamyloid oligomers and nuclei, respectively (scale bar, 5μm). Confocal images demonstrate that there was no time-dependent increase in oligomers when incubated under control conditions for 120min, in contrast to atrial HL-1 cells exposed to IsoLGs (Figure 3B).

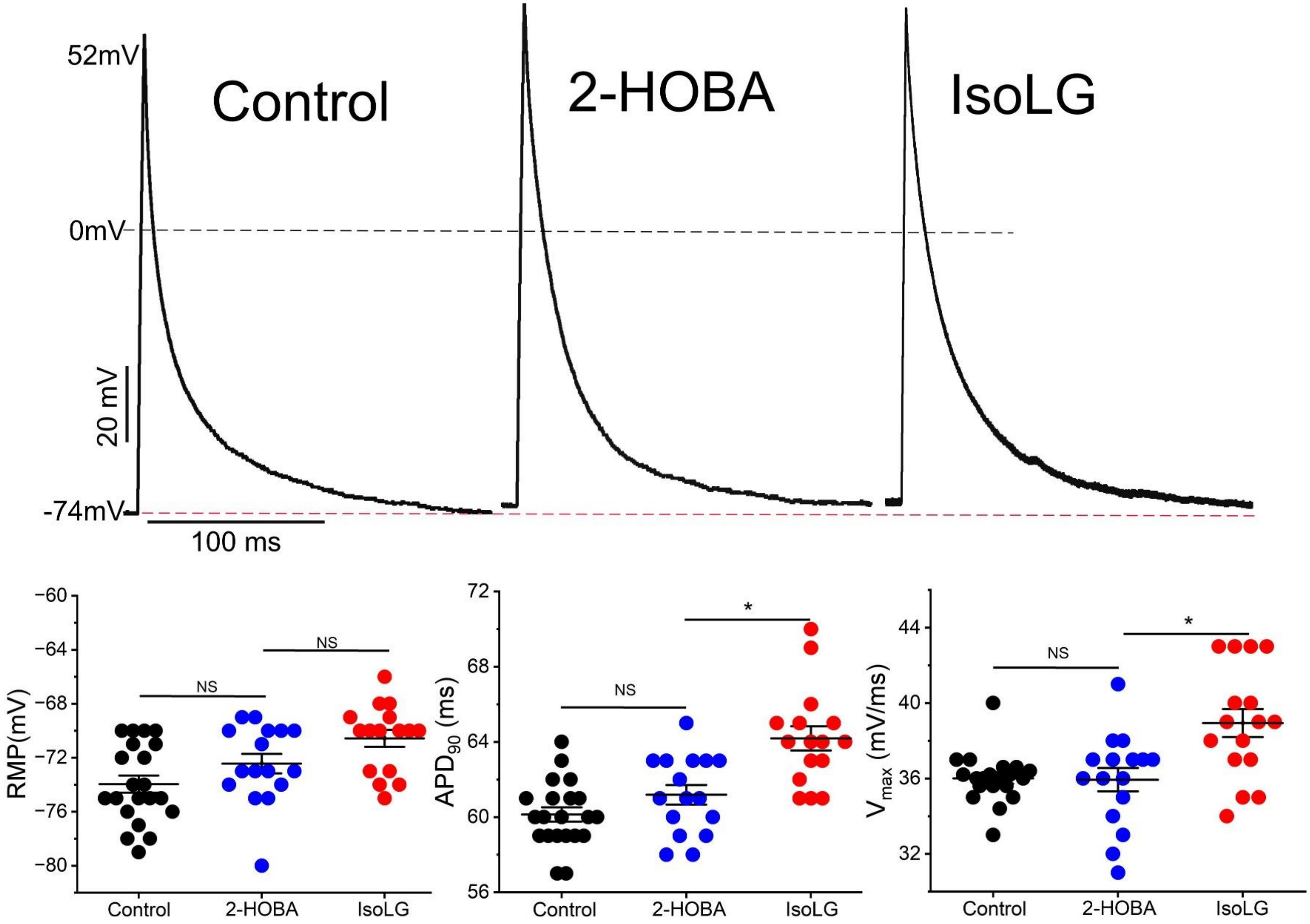

Figure S2: Preincubation of mouse atrial cardiomyocytes with 2-hydroxybenzylamine (2-HOBA) prevents isolevuglandin (IsoLG) effects. Mouse atrial cells were preincubated in 2-HOBA 100µM for 30 min and action potentials (APs) recorded at 1Hz, followed by exposure to IsoLG 500nM and repeated recordings. Control data represent historical controls for comparison. The red dashed line is the resting membrane potential under control conditions and the black dashed line represents 0mV. The values for resting membrane potential and AP overshoot under control conditions are shown. AP amplitudes were 126mV, 132mV, and 131mV, respectively, for the 3 APs. After exposure to 2-HOBA, IsoLGs did not cause a significant change in resting membrane potential (RMP), action potential duration (APD90) and maximum phase 0 upstroke slope (Vmax) (n=21, 16, 16 for control, 2-HOBA, and 2-HOBA plus IsoLGs, respectively; ANOVA followed by unpaired t test). 2-HOBA prevented the reduction in APD and Vmax typically caused by the addition of IsoLGs (paired t test following ANOVA). *P<0.05.

II. SUPPLEMENTARY TABLES

Table S1: Effects of IsoLGs on action potentials from mouse atrial myocytes

	RMP mV	APD_{90} ms	APD_{50} ms	V_{max} mV/ms
Control	-74.0±0.6	60.1±0.4	22.3±0.4	36.0±0.3
IsoLGs 500 nM	-71.1±0.9***	58.1±0.4***	21.1±0.4***	35.5±0.3***

***P<0.001; n=21 for each

Table S2: Effects of IsoLGs on action potentials from atrial HL-1 cells

	RMP mV	APD_{90} ms	APD_{50} ms	V_{max} mV/ms
Control	-75.4±0.9	59.4±0.4	22.7±0.7	32.2±0.7
IsoLGs 500 nM	-73.5±0.9***	56.9±0.5***	21.0±0.7***	30.7±0.7***

***P<0.01; n=26 for each

Table S3: IsoLG effects after preincubation with 2-HOBA in mouse atrial myocytes

	RMP mV	APD_{90} ms	APD_{50} ms	V_{max} mV/ms
Control	-74.0±0.6	60.1±0.4	22.3±0.4	36.0±0.3
2-HOBA 100µM	-72.4±0.7	61.2±0.5	22.4±0.4	35.9±0.6
IsoLG 500 nM	-70.3±0.7	64.3±0.7*	23.7±0.4	38.9±0.7*

*P<0.05; n=21, 16, 16

Control data represent historical controls; after ANOVA, control vs 2-HOBA with unpaired t test; 2-HOBA vs 2-HOBA + IsoLG with paired t test

Table S4: IsoLG effects after preincubation with 2-HOBA in atrial HL-1 cells

	RMP mV	APD_{90} ms	APD_{50} ms	V_{max} mV/ms
Control	-75.4±0.9	59.4±0.4	22.7±0.7	32.2±0.7
2-HOBA 100µM	-72.2±0.6*	57.9±0.5*	19.2±0.3*	31.2±0.4
IsoLG 500 nM	-70.0±0.8	61.3±0.4	21.1±0.5	36.7±0.6*

*P<0.05; n=26, 15, 15

Control data represent historical controls; after ANOVA, control vs 2-HOBA with unpaired t test; 2-HOBA vs 2-HOBA + IsoLG with paired t test

Table S5: Effects on the voltage dependence of ion channel activation

	I_{Na}	$I_{Ca,L}$	I_{sus}	I_{To}
Control	-40.7±0.5	4.1±0.1	11.6±1.9	36.2±0.3
IsoLG 500 nM	-39.0±0.5***	6.1±0.2***	9.0±1.7**	33.7±0.3**

All data (in mV) are expressed as the midpoint ($V_{1/2}$ or half-maximum) of the conductance (or tail current for I_{sus})-voltage activation curve

**P<0.01, ***P<0.001; n=11-16

Table S6: Effects on ion current amplitude

	I_{Na} (pA/pF)	$I_{Ca,L}$ (pA/pF)	I_{sus} (pA/pF)	I_{To} (pA/pF)
Control	-74.0±3.5	-29.4±3.3	50.8±4.7	56.8±1.4
IsoLG 500 nM	-59.0±3.1***	-21.9±2.4***	55.6±5.1**	68.5±1.6**

**P<0.01, ***P<0.001; n=11-16

Table S7: Primers used in the qRT-PCR experiments.

Gene	Forward(5'→3')	Reverse(5'→3')
mtDNA	CTAGAAACCCCGAAACCAAA	CCAGCTATCACCAAGCTCGT
B2m	ATGGGAAGCCGAACATACTG	CAGTCTCAGTGGGGGTGAAT
Ppargc1a	TTCGGGAGCTGGATGGCTTG	CAGGAAGATCTGGGCAAAGAGG
Nrf	AGAAACGGAAACGGCCTCAT	ACTCATCCAACGTGGCTCTG
Tfam	CCCGGCAGAGACGGTTAAAA	TCCCTGAGCCGAATCATCCT
Ndusf4	AACCCAAGTCCAAGTCTTATG	CACAGTCAAGCAGAGATGTAG
Sod1	GGGCAAAGGTGGAAATGA	CCACACAGGGAATGTTTAC
Sod2	CCACCGAGGAGAAGTACCAC	CTCCAGCAACTCTCCTTTGG
Gpx1	CAGAGTGCAGCCAGTAATC	CCAGGAGAATGGCAAGAATG
Gapdh	TGCCAAGTATGATGACATCAACAAG	AGCCCAGGATGCCCTTTAGT

III. SUPPLEMENTARY METHODS

Compounds, reagents, and probes

15-E_2-isolevuglandin (IsoLG) was synthesized as described[1] and stored at -80°C until use. Oligomycin, carbonyl cyanide p-trifluoromethoxyphenylhydrazone (FCCP), rotenone, and antimycin A were obtained from Sigma-Aldrich (St. Louis, MO). Dihydroethidium (DHE), MitoSOX™ Red, tetramethylrhodamine methyl ester (TMRM), Hoechst 33342 and Mitotracker Green were purchased from Thermo Fisher Scientific (Waltham, WA). 2-Hydroxybenzylamine (2-HOBA) acetate was provided by Metabolic Technologies, Inc. (Missoula, MT).

Mouse atrial cardiomyocyte isolation

Male C57BL/6J mice were obtained from Jackson Laboratory and studied at 3 to 4 months of age. Mice were maintained under 12:12h light-dark cycles with standard chow and water *ad libitum*. All animal procedures were approved by the Vanderbilt Institutional Animal Care and Use Committee with care in accordance with the Guide for the Care and Use of Laboratory Animals, US Department of Health and Human Services. Mouse atrial cardiomyocytes were isolated using collagenase digestion.[2] Mice were injected with heparin (100U/ per mouse) intraperitoneally and anesthetized with isoflurane (3-4%) inhalation. A midline thoracotomy was performed, the heart was excised, quickly cannulated via the aorta, mounted to a Langendorff apparatus, and perfused with a Ca^{2+}-free perfusion buffer containing (in mM) 113 NaCl, 4.7 KCl, 0.6 KH_2PO_4 , 1.2 $MgSO_4$, 5.5 glucose, 0.6 Na_2HPO_4, 12 $NaHCO_3$,

10 $KHCO_3$, 10 HEPES, 10 2,3-butanedione monoxime (BDM), and 30 taurine at a rate of 3ml/min for 4min at 37°C. Thereafter, the heart was perfused with a digestion buffer containing collagenase II (Worthington; 300U/ml) and 12.5µM $CaCl_2$, for 8-12min at 37ºC, until the heart became pale and flaccid. The heart was removed, and the atria were dissected, gently teased into small pieces with fine-tip forceps, and further dissociated in digestion buffer with a wide-bore (2mm) Pasteur pipette for 5min. Digestion was terminated with a buffer containing 10% FBS and 12.5µM $CaCl_2$, and the atrial cardiomyocyte suspension was filtered through 200µm mesh and allowed to sediment by gravity for 20min at room temperature. The final atrial cardiomyocyte pellet was resuspended in perfusion buffer and Ca^{2+} reintroduction was performed in 5 steps to 1mM.

Cell culture

Atrial HL-1 cells were obtained from Dr. William Claycomb[3] and grown on 10 cm culture dishes pre-coated with fibronectin/gelatin under a 5% CO_2 atmosphere in Claycomb Medium supplemented with 10% fetal bovine serum (FBS), 0.1mM norepinephrine, 2mM L-glutamine (all from Sigma-Aldrich), as described previously.[4] Cells were seeded into either 96-well or 6-well plates, or chamber slides and incubated overnight at 37ºC, 5% CO_2 in an incubator. The next day, Claycomb Medium was removed, and the cells were washed 2 times with 1xPBS and incubated with IsoLG in Hanks Balanced Salt Solution (HBSS; Gibco) for 2h using IsoLG concentrations indicated in the figure legends. After treatment, HBSS was removed from the wells, and residual IsoLG neutralized by incubating cells with 1% FBS in HBSS for 5min prior to use. Mouse fibroblast (Ltk^-) cells that stably express *KCNA5*, representing the rapidly-activating sustained outward K^+ current I_{sus}, were also cultured as described.[2]

Cytotoxicity

Atrial HL-1 cells were plated at density of 25,000 cell per well in 100µl Claycomb Medium in a 96-well microplate (Perkin Elmer, Waltham, MA) precoated with gelatin and fibronectin and incubated overnight (37°C, 5% CO_2). The next day, Claycomb Medium was replaced with HBSS and cells were treated with different concentrations of IsoLGs for 2h. Cytotoxicity of IsoLGs was determined by measuring cellular ATP levels with an ATPlite assay (Perkin Elmer, Waltham, MA) according to the manufacturer's instructions. Luminescence was measured using a Lumicount microplate reader (Global Medical Instrumentation, Ramsey, MN).[5]

Metabolic flux assays

The Seahorse XFp Cell Mito Stress Test (Agilent) was used to analyze oxygen consumption rates (OCRs) and other bioenergetic parameters in atrial HL-1 cells in response to IsoLG treatment.[2] Briefly, HL-1 cells were seeded and cultured in Seahorse XFp tissue culture microplates at a density of 1.5×10^4 cells per well in 80µL of Claycomb media overnight. The next day, the medium was exchanged with HBSS and cells were incubated with different concentrations of IsoLG for 2h, with the reaction terminated by 1% FBS in HBSS. Thereafter, the cells were washed 3 times with Seahorse base medium supplemented with 1mM pyruvate, 2mM glutamine and 5.5mM glucose. Cells were then incubated in Seahorse base medium in a non-CO_2 incubator at 37°C for 1h and OCR was measured with a Seahorse XFp Analyzer following sequential injections of oligomycin (1µM), FCCP (1µM), and antimycin A/rotenone (1µM). Cells were then stained with Hoechst33342 for cell number normalization and imaged

with ImageXpress Micro XLS System (Molecular Devices). Subsequently, cell number in each well was determined with MetaXpress High Content Image Acquisition & Analysis Software (Molecular Devices) employing the Multi Wavelength Cell Scoring function. Cell numbers were then used to normalize all OCR parameters. The final OCR values were calculated by averaging the results of at least 3 biologically independent experiments using Multi-File XFp Cell Mito Stress Test Report Generator (Agilent).

Measurement of mitochondrial membrane potential (Δψm)

Atrial HL-1 cells were plated in either 96-well microplates ($1.5x10^4$ cells/well, Greiner) or 8-well chamber slides ($4x10^4$ cells/well, Ibidi) for 24h. Claycomb Medium was exchanged with HBSS medium following extensive washing with 1x phosphate buffered saline (PBS), and cells were treated with IsoLG for 2h at a range of concentrations. After IsoLG treatment, cells were washed twice with 1xPBS and incubated with TMRM (50nM), Mitotracker Green (100nM) and Hoechst 33342 (5µM) for 30min at 37°C, 5% CO_2 in an incubator. As a positive control, a cohort of cells was treated with FCCP (20µM) for 30min prior to incubation with TMRM. Cells were washed, and live cell images were obtained using either ImageXpress Micro XLS System (Molecular Devices) for high content analysis or using a Zeiss 880 confocal microscope (Carl Zeiss Microimaging, Inc., Oberkochen, Germany).

Mitochondrial DNA (mtDNA) content

Total DNA was isolated from HL-1 cells as described previously.[6] mtDNA content was calculated from the ratio of mtDNA to nuclear DNA (*B2m*) by quantitative polymerase chain

reaction (qPCR).[7] Primer sequences for mtDNA are displayed in **Table S7**. Data analysis was performed using the $2^{-\Delta\Delta CT}$ method.[8]

Quantitative reverse transcription-polymerase chain reaction (qRT-PCR)

Total RNA from atrial HL-1 cells was isolated using Trizol reagent (Thermo Fisher Scientific, Waltham, MA). First strand cDNA synthesis was performed using the Applied Biosystems High-Capacity cDNA Reverse Transcription Kit (Thermo Fisher Scientific, Waltham, MA) starting from 1μg total RNA. qRT-PCR was performed using PowerUp SYBR Green Master Mix (Thermo Fisher Scientific, Waltham, MA) in 20µl reactions on an CFX96 Touch Real-Time PCR Detection System. In all experiments, *Gapdh* was used for normalization. Data analysis was performed using the $2^{-\Delta\Delta CT}$ method.[8] Primer sequences used are shown in the **Table S7** of the **Supplementary Materials**.

Detection of cytosolic $O_2^{\bullet-}$ ***production***

The fluorescent dye dihydroethidium (DHE) was employed to assess intracellular $O_2^{\bullet-}$ production in atrial HL-1 cells in response to IsoLG treatment. Briefly, cells were seeded in Ibidi 8-well glass chamber slides and allowed to attach overnight. On the day of the experiment, cells were incubated with IsoLG in HBSS for 2h. Cells were washed, IsoLG was neutralized with 1% FBS in HBSS, and cells were incubated in HBBS containing 10μM DHE for 30min at 37°C, 5% CO_2 in an incubator. Excess DHE was washed away, and live cell images were acquired with a Zeiss 880 confocal microscope.

Detection of mitochondrial $O_2^{\bullet-}$ ***production***

After treatment with IsoLG or 2-HOBA, HL-1 cells were washed with 1xPBS prior to incubation with 2μM MitoSOX Red in HBSS for 30min at 37°C, 5%CO_2 in an incubator. Cells were washed with HBSS briefly and live cell images were captured with a Zeiss 880 confocal microscope.

Protein carbonylation

An OxiSelect™ Protein Carbonyl Immunoblot Kit (Cell Biolabs Inc., San Diego, CA) was used to analyze carbonylated proteins according to the manufacturer's instructions. Briefly, cells cultured in 6-well plates were treated with IsoLG in HBSS for 2h. Protein extraction was performed using RIPA lysis buffer (Sigma-Aldrich, Saint Louis, MO) that included phosphatase and protease inhibitors. Protein concentrations were measured using the BCA assay and protein lysates were separated on 4-15% pre-cast Novex gels and blotted on a polyvinylidene fluoride (PVDF) membrane followed by pretreatment with methanol and 2N HCl, and post-electrophoresis derivatization with dinitrophenylhydrazine (DNPH) for 5min at room temperature. Membranes were blocked for 1h in 5% nonfat dry milk and incubated with a rabbit anti-DNP antibody (1:1000) overnight at 4°C. Thereafter, membranes were washed and incubated with IRDye secondary antibodies (LI-COR) with detection using a LI-COR Odyssey Infrared Imaging System (LI-COR Biosciences, Lincoln, NE). β-actin (1:500; Santa Cruz Biotechnology, Dallas, TX) was used as an internal loading control.

Preamyloid oligomer (PAO) formation

PAO formation was assayed in atrial HL-1 myocytes following IsoLG stimulation as described previously,[9] with minor modifications. Briefly, cells were grown on glass coverslips

and exposed to IsoLG (0.1µM) for various time periods (0h, 0.25h, 0.5h, 1h, 1.5h, and 2h). Cells were washed with HBSS containing 1% FBS, fixed with 4% paraformaldehyde (PFA) in 1xPBS for 10min and permeabilized with 0.1% Triton in 1xPBS for 5min. Unreacted aldehyde groups were quenched with 0.1M glycine and non-specific binding sites were blocked with Power Block (BioGenex, San Ramon, CA) for 1h. Cells were incubated with a conformation-specific, polyclonal rabbit A-11 antibody (1:3000, EMD Millipore) overnight at 4°C. The next day, coverslips were washed with PBS and incubated with rabbit Alexa Fluor 488 antibody for 1h in the dark. Subsequently, cells were washed and mounted on glass slides with Prolong Gold antifade medium, cured overnight in the dark and imaged with a Zeiss 880 confocal microscope.

Apoptosis

Apoptotic activity in response to IsoLG treatment was monitored using CellEvent™ Caspase-3/7 Green Detection Reagent (Thermo Fisher Scientific, Waltham, MA) according to manufacturer's instructions. Briefly, cells were seeded in Ibidi chamber slides and cultured overnight. On the day of the experiment, cells were incubated with HBSS containing IsoLG for 2h. IsoLG was neutralized with 1% FBS, and cells were incubated with CellEvent™ Caspase-3/7 Green Detection Reagent for 30min, washed, stained with Hoechst, and imaged with a Zeiss 880 confocal microscope using identical confocal settings (e.g., laser intensity, detector gain, and pinhole size, etc).

Quantitation of oxidative phosphorylation proteins

Following IsoLG treatment, cells were washed with cold PBS twice and protein extraction was performed using RIPA lysis buffer (Sigma-Aldrich, Saint Louis, MO) containing

phosphatase and protease inhibitors (Roche Diagnostics, Indianapolis, IN). Protein concentrations were measured using the BCA assay (Pierce) and protein lysates were separated on 4-15% pre-cast Novex gels and blotted on a nitrocellulose membrane. Membranes were blocked for 1h in 5% nonfat dried milk (Bio-Rad, Hercules, CA) and then incubated with total oxidation phosphorylation (OXPHOS) rodent antibody cocktail (1:1000; Abcam, Waltham, MA) or β-actin (1:500, Santa Cruz Biotechnology, Dallas, TX) overnight at 4°C. Thereafter, membranes were washed and incubated with IRDye secondary antibodies for 1h in the dark. The membranes were visualized with a LI-COR Odyssey Clx Infrared Imaging System and band intensities were analyzed with Image Studio Lite (LI-COR Biosciences, Lincoln, NE).

Electrophysiology

The whole cell configuration was used to record action potentials and ionic currents from single cells as described previously.[2] Data were acquired using the current or voltage clamp mode of an Axopatch 200B amplifier (Molecular Devices, Sunnyvale, CA) connected to a Digidata 1320A interface (Molecular Devices, Sunnyvale, CA). Pipettes were pulled (Sutter Instrument, Novato, CA) using capillary tubes (Micro-Hematocrit Capillary Tubes Cat #:AF-22-362-574; Thermo-Fisher Scientific, Waltham, MA) with tip resistances of 0.8 to 1 MΩ. Currents were low-pass filtered at 5 kHz using an Axopatch 200B amplifier and digitized at 10 kHz with a Digidata 1320A A/D converter. Capacitance and 80–95% series resistance was routinely compensated.

Action potentials. Action potentials were recorded during stimulation at 1Hz from mouse atrial myocytes or atrial HL-1 cells at 37°C using the current clamp technique as described previously.[2] The extracellular Tyrodes's solution was bubbled with 100% O_2 and

contained (in mM): NaCl 145, KCl 4, $MgCl_2$ 1, $CaCl_2$ 1.8, and HEPES 10 (pH 7.4). The pipette-filling solution contained (in mM): K-DL-aspartate 120, KCl 25, $MgCl_2$ 1, EGTA 10, Na_2phosphocreatine 2, Na_2ATP 4, NaGTP 2, and HEPES 5 (pH 7.2). Action potentials were triggered using a current stimulus of 4ms duration and 250-500pA amplitude. Cells were selected for experimentation if the resting membrane potential was below -55mV and the overshoot exceeded 20mV.

Ionic currents. The whole cell configuration was used to record ionic currents from atrial HL-1 cells (I_{Na} and I_{To}), *Ltk*⁻ cells (I_{sus}, represented by KCNA5 current), and mouse atrial myocytes ($I_{Ca,L}$) at room temperature (22±1°C). To calculate ionic current conductance:

$G_i = \frac{I_{ion}}{(Vm - E_{rev})}$ where, Gi is conductance, I_{ion} is ionic current, and (V_m-E_{rev}) is the driving force, where V_m is the conditioning voltage and E_{rev} is the reversal potential of the ion. All activation and inactivation curves were fitted with a single Boltzmann function as follows:

$y = A2+ (A1-A2)/ (1+\exp((V_m - V_{1/2})/k))$ where A1 is maximum current, A2 is the minimum current, $V_{1/2}$ is the half-activation potential or half-inactivation potential (i.e., half availability), Vm is test voltage, and k is slope factor.

To record I_{Na}. To record Na^+ currents, the holding potential was -120mV. The pipette solution contained the following (in mM): NaF 10, CsF 110, CsCl 20, EGTA 10, and HEPES 10, pH 7.2. The extracellular solution was composed of (in mM): NaCl 10, CsCl 110, TEA-Cl 5, $CaCl_2$ 0.1, $MgCl_2$ 1, HEPES 10, and glucose 10, pH 7.4. To eliminate L- and T-type calcium currents, nimodipine 2µM and mibefradil 10µM were added to the bath solution. The voltage dependence of channel activation was measured using 100ms test pulses from -90mV to +60mV in 10mV increments. To assay the voltage-dependence of steady-state inactivation, the

membrane potential was stepped from -160mV to -10mV in 10mV increments (200ms pulses) using a holding potential of -100mV, followed by 40ms test pulse to -20mV.

To record $I_{Ca,L}$. To record $I_{Ca,L}$, the pipette solution consisted of (in mM): Cs-aspartate 120, EGTA-CsOH 10, tetraethylammonium chloride (TEA-Cl) 10, $CaCl_2$ 3, Mg_2ATP 1, phosphocreatine 5, NaGTP 1, and 10 HEPES, pH 7.2. The bath solution contained (in mM): TEA-Cl 140, $MgCl_2$ 2, $CaCl_2$ 5, glucose 10, and HEPES 10, pH 7.4. A holding potential of -40mV was used, and the cycle time for pulse protocols was 5sec. Voltage was sequentially stepped for 300ms from -40mV to +70mV in 10mV increments.

To record I_{To}. For I_{To}, the pipette solution consisted of (in mM): K-DL-aspartate 120, KCl 25, $MgCl_2$ 1, $CaCl_2$ 1, EGTA 10, HEPES 5, Na_2phosphocreatine 2, NaATP 4, and NaGTP 2, pH 7.2. The bath solution contained (in mM): NMDG 160, KCl 4, $MgCl_2$ 1, Glucose 10, and HEPES 10, pH 7.4. A holding potential of -70mV was used and the cycle time for pulse protocols was 2sec. Voltage was sequentially stepped for 500ms from -40mV to +60mV in 10mV increments.

To record I_{sus}. To record K^+ currents from *KCNA5*-transfected *Ltk*$^-$ cells, the pipette solution consisted of (in mM): K-DL-aspartate 120, KCl 25, $MgCl_2$ 1, Na_2 phosphocreatine 2, EGTA 10, Na_2ATP 4, NaGTP 2, and HEPES 5, pH 7.2. The bath solution was Tyrode solution with (in mM): NaCl 145, KCl 4, $MgCl_2$ 1, $CaCl_2$ 1.8, and HEPES 10, pH 7.4. A holding potential of -80mV was used and the cycle time for pulse protocols was 2sec. Test potentials (100ms) were stepped from -100mV to +60mV, with tail currents recorded upon repolarization to -30mV for 100ms.

Statistics

Data are presented as mean ± SEM. Statistical significance was determined by one-way ANOVA and *post hoc* Tukey test for multiple comparisons using Graph Pad Prism software (Version 9.1.2) unless specified otherwise in the figure legends. Differences with a P value < 0.05 were considered significant. For electrophysiologic recordings, data were analyzed using Clampfit 10.0 software (Molecular Devices, Sunnyvale, CA), compiled in Excel Office 365 (Microsoft, Redmond, WA), and plotted and fitted in OriginPro 2022 (OriginLab Corporation, Northampton, MA). Unless specified, Student's paired t test (two-tailed) was used when appropriate.

REFERENCES

1. Amarnath V, Amarnath K, Masterson TS, Davies S and Roberts LJ. A simplified synthesis of the diasteromers of levuglandin E2. *Synthetic Communications*. 2005;35:397-408.

2. Yang Z, Subati T, Kim K, Murphy MB, Dougherty OP, Christopher IL, Van Amburg JC, Woodall KK, Barnett JV and Murray KT. Natriuretic Peptide Oligomers Cause Proarrhythmic Metabolic and Electrophysiological Effects in Atrial Myocytes. *Circ Arrhythm Electrophysiol*. 2022;15:e010636.

3. Claycomb WC, Lanson NA, Jr., Stallworth BS, Egeland DB, Delcarpio JB, Bahinski A and Izzo NJ, Jr. HL-1 cells: A cardiac muscle cell line that contracts and retains phenotypic characteristics of the adult cardiomyocyte. *Proceedings of the National Academy of Sciences of the United States of America*. 1998;95:2979-2984.

4. Yang Z, Shen W, Rottman JN, Wikswo JP and Murray KT. Rapid stimulation causes electrical remodeling in cultured atrial myocytes. *J Mol Cell Cardiol*. 2005;38:299-308.

5. Prinsen JK, Kannankeril PJ, Sidorova TN, Yermalitskaya LV, Boutaud O, Zagol-Ikapitte I, Barnett JV, Murphy MB, Subati T, Stark JM, Christopher IL, Jafarian-Kerman SR, Saleh MA, Norlander AE, Loperena R, Atkinson JB, Fogo AB, Luther JM, Amarnath V, Davies SS, Kirabo A, Madhur MS, Harrison DG and Murray KT. Highly-reactive isolevuglandins promote atrial fibrillation caused by hypertension. *JACC Basic Transl Sci*. 2020;5:602-615.

6. Miller SA, Dykes DD and Polesky HF. A simple salting out procedure for extracting DNA from human nucleated cells. *Nucleic Acids Res*. 1988;16:1215.

7. Malik AN, Czajka A and Cunningham P. Accurate quantification of mouse mitochondrial DNA without co-amplification of nuclear mitochondrial insertion sequences. *Mitochondrion*. 2016;29:59-64.

8. Livak KJ and Schmittgen TD. Analysis of relative gene expression data using real-time quantitative PCR and the 2(-Delta Delta C(T)) Method. *Methods*. 2001;25:402-408.

9. Sidorova TN, Yermalitskaya LV, Mace LC, Wells KS, Boutaud O, Prinsen JK, Davies SS, Roberts LJ, 2nd, Dikalov SI, Glabe CG, Amarnath V, Barnett JV and Murray KT. Reactive gamma-ketoaldehydes promote protein misfolding and preamyloid oligomer formation in rapidly-activated atrial cells. *J Mol Cell Cardiol*. 2015;79:295-302.
